# Predicting Daily Aerobiological Risk Level of Potato Late Blight Using C5.0 and Random Forest Algorithms under Field Conditions

**DOI:** 10.3390/s23083818

**Published:** 2023-04-08

**Authors:** Laura Meno, Olga Escuredo, Isaac K. Abuley, M. Carmen Seijo

**Affiliations:** 1 Department of Vegetal Biology and Soil Sciences, Faculty of Sciences, University of Vigo, 32004 Ourense, Spain; 2 Department of Agroecology, Flakkebjerg Research Center, Aarhus University, Forsøgsvej 1, 4200 Aarhus, Denmark

**Keywords:** aerobiology, *Solanum tuberosum* L., *Phytophthora infestans*, weather factors, infection pressure, machine learning

## Abstract

Late blight, caused by *Phytophthora infestans*, is a major disease of the potato crop with a strong negative impact on tuber yield and tuber quality. The control of late blight in conventional potato production systems is often through weekly application of prophylactic fungicides, moving away from a sustainable production system. In support of integrated pest management practices, machine learning algorithms were proposed as tools to forecast aerobiological risk level (ARL) of *Phytophthora infestans* (>10 sporangia/m^3^) as inoculum to new infections. For this, meteorological and aerobiological data were monitored during five potato crop seasons in Galicia (northwest Spain). Mild temperatures (T) and high relative humidity (RH) were predominant during the foliar development (FD), coinciding with higher presence of sporangia in this phenological stage. The infection pressure (IP), wind, escape or leaf wetness (LW) of the same day also were significantly correlated with sporangia according to Spearman’s correlation test. ML algorithms such as random forest (RF) and C5.0 decision tree (C5.0) were successfully used to predict daily sporangia levels, with an accuracy of the models of 87% and 85%, respectively. Currently, existing late blight forecasting systems assume a constant presence of critical inoculum. Therefore, ML algorithms offer the possibility of predicting critical levels of *Phytophthora infestans* concentration. The inclusion of this type of information in forecasting systems would increase the exactitude in the estimation of the sporangia of this potato pathogen.

## 1. Introduction

Late blight, caused by *Phytophthora infestans*, is a major disease of potato crop, with a strong negative impact on tuber yield and quality [1,2,3]. The pathogen is regarded as a threat to global food security because worldwide losses due to late blight are estimated to exceed annually $5 billion [4,5]. *P. infestans* is described as a lower water oomycete and infects the potato crop through the tuber and soil during cool and wet weather. Infection of shoots can be caused by mycelium growing from the tuber into the developing shoot or through sporangia and zoospores formed on the tuber surface under wet conditions [6]. Then, the potential risk of disease development depends in part on the aerial transport of *P. infestans* sporangia to potato fields from neighbor’s infection fields [7,8,9,10]. Depending on host susceptibility and environmental conditions, the first symptoms can be visible 3–4 days after infection [1]. Night temperatures of 10–16 °C accompanied by light rain, fog or heavy dew and followed by days of 13–16 °C with high relative humidity are ideal conditions for late blight infection and development [11]. The first symptoms are followed by the production of new sporangia and the infection cycle is repeated as many times as the weather conditions allow the viability of the released sporangia [6,8,12,13].

Potato crops contributed to alleviating world hunger and potatoes remain one of the agricultural resources needed in line with the zero-hunger sustainable development goal. However, the crop management strategies increasingly require the application of phytochemicals, which may cause undesirable effects on the environment and on human health, with an increasing production costs for growers. For effective and ecofriendly management of potato late blight, scientific and technical efforts must be made to understand how the disease progresses and how this progression can be slowed down. One of the important issues in late blight management is to forecast when, where and how abundant airborne inoculum will be, to prevent the onset of the epidemic. The airborne inoculum of the pathogen appears to have a significant impact on the disease epidemic. However, the prediction of airborne spores of plant pathogens is difficult because they are influenced by a plethora of factors (temperature, relative humidity, leaf wetness, wind, phenological stage) [7,10,12,14,15]. Hence, the efforts towards understanding and predicting airborne sporangia of *P. infestans* based on multiple factors that condition its development, with a significant impact on the management of late blight, with less fungicide applications are sought.

In recent years, the agricultural sector was able to adopt the main technological innovations relying on artificial intelligence (AI), artificial neural networks (NN) and machine learning (ML). The goal is to digitize itself and increase the autonomy of many processes by making better data-driven decisions, reducing the workload, inputs and increase the quality of the final product [16,17,18,19,20]. The multi-view spectral information from unmanned aerial vehicles (UAV) based color-infrared images combined with machine learning algorithms was used to improve the estimation of nitrogen nutrition status in winter wheat and optimize the fertilization [17]. Classification methods and clustering trough image analyses such as neural networks (CNN) were used to simulate the humans’ decision-making process. CNNs were shown to have great potential for fine classification problems using an image of the same object from different views [18,19]. Decision trees, support vector machines or k-means together with information from foliage of the crop were used in precision agriculture and the effective detection, identification and quantification of plant diseases [21,22]. In the case of potato crop, ML algorithms were recently applied for monitoring diseases through image-based techniques [20,23,24,25,26,27]. Sugiura et al. [24] proposed a phenotyping system for mapping late blight on potato crop by analyzing pixel change between consecutive images. The assessment of late blight severity in potato by acquiring high resolution multispectral images with a low-cost camera and ML algorithms was also reported [25]. More recently, the early detection and severity assessment of late blight in potato crops by multispectral imagine were evaluated [26,27]. However, these studies focused on the detection and identification of disease after the onset of the infection process. The preceding step is the early detection of inoculum in the environment of the crop able to cause first late blight symptoms on the potato canopy. Aerobiology is an excellent discipline for this purpose, allowing real time knowledge of sporangia in the potato atmosphere [9,10,14,15,28,29,30].

There were some aerobiological studies that focused on understanding the influence of climatic factors on the dynamics of spores in the atmosphere of the potato crop using different multivariate statistical techniques and ML algorithms [31,32,33]. However, there were fewer studies trying to predict *P. infestans* sporangia levels in the environment crop [10,15]. Furthermore, despite the great available scientific information on late blight, few studies focused on the specific value of airborne sporangia concentration as a monitoring tool for late blight control [10,14,15,29,34,35]. In this sense, with the purpose of estimating the late blight risk during the early stages of potato crop development, ML algorithms were applied. The goals of the present study were: (i) to assess the concentration of *P. infestans* in each phenological stage of potato crop in northwest Spain; (ii) to derive a simple binary classification model for predicting the days exceeding the aerobiological risk level of pathogen; and (iii) to validate ML algorithms as a tool for forecasting late blight outbreaks.

## 2. Materials and Methods

### 2.1. General Aspects of the Experimental Potato Field

The experimental field was located in A Limia (Galicia, northwest Spain) and the study was conducted over five crop seasons (2017–2021). A 4-hectare field was planted with the potato cultivar Agria, considered as a medium resistant to late blight disease. The field plot was managed under an annual wheat–potato rotation system. The dates of planting and the main phenological stages for each season are shown in Table 1. The phenological monitoring started when 50% of plants emerged, and weekly observations until crop senescence were performed. Three main phenological stages considering the BBCH scale of Hack et al. [36] with some modifications [37] were monitored: foliar development (FD), flowering (FL), senescence (SE).

### 2.2. Weather Monitoring

Weather data were registered hourly using a portable weather station i-METOS 3.3. (Pessl Instruments, Weiz, Austria) placed in the middle of the experimental potato field, at 1.5 m height since 50% of plants were emerged. Daily mean temperature (T, °C), relative humidity (RH, %), leaf wetness (LW, h) and wind speed (Wind, m/s) were calculated with hourly data registered. 

The release of sporangia from the sporangiophore (expressed as spore release, SR) was calculated using hourly RH and a critical value of 88% of RH [7]. The escape of sporangia from the canopy into the atmosphere (escape) was calculated with the hourly wind speed data according to the formula proposed by Skelsey et al. [7].

Following the Danish late blight model (BlightManager), the daily risk value (DRV) based on hourly temperature and relative humidity was calculated [38]. Subsequently, the infection pressure (IP), which is a running sum of the DRV of five days, was calculated as described previously [10,38].

### 2.3. Aerobiological Sampling

The surveillance of the atmosphere of the crop was performed through an aerobiological sampler type Lanzoni VPPS 2000 (Lanzoni S.r.l., Bologna, Italy) placed in the potato plot at 1.5 m high close to the weather station since the 50% of plants emerged. The equipment contained a vacuum pump to aspirate the airborne particles surrounding through a horizontal aperture, which was connected with the melinex tape covered where these particles were retained. A clockwork-driven drum containing the tape was moving continuously for 7 days, and each week, the tape was replaced. The methodology used for the assembly and counting of airborne particles was based on the proposal by Galán et al. [39], expressing the concentration of *P. infestans* in sporangia/m^3^.

**Figure 1:**
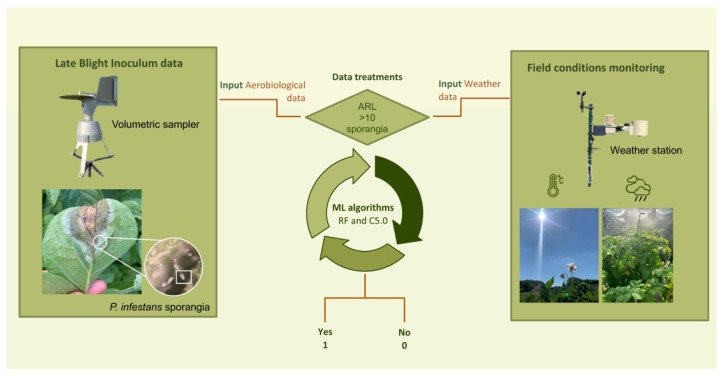
Visual abstract about the inputs (weather and aerobiological variables) used in the machine learning (ML) algorithms. ARL: daily aerobiological risk level.

Based on field experiences, a daily aerobiological risk level (ARL) of 10 sporangia/m^3^ was established. This level defines the concentration of sporangia likely to cause late blight infection in the potato crop, considered in the statistical treatment of ML algorithms (Figure 1).

### 2.4. Data Analyses

Data curation and statistical analyses were performed with the R language and environment for statistical computing version 4.1.3 [40] and IBM SPSS Statistics 22 program. Spearman correlation test was applied to analyze the relationships between weather factors and presence of sporangia until seven previous days, with a significance level of *p* < 0.05.

Prior to the treatment of ML algorithms, a binary code based on the ARL established by the aerobiological method explained above was proposed. The code established the zero (0) value for days with a concentration lower than 10 sporangia/m^3^, while the days that exceeded this concentration threshold were classified as 1 (Figure 1). Then, ML algorithms C5.0 and random forest (RF) to predict ARL were applied. The C5.0 algorithm is a decision tree ML algorithm that uses entropy (a measure of the randomness in a partition) for splitting trees. The C5.0 model uses adaptive boosting to improve the predictive power of the final model. The RF algorithms, also known as decision tree ensemble algorithms, combine the results of multiple independent decision trees to make predictions about new data sets [41]. Each tree in the forest assigns the most probable class label to each input.

#### Implementing the Machine Learning Algorithms

The data set was split into 80% (469 values/rows) training and 20% (117 values/rows) test data sets. As the tested algorithms were not based on distance metrics, no standardization was carried for the data. These algorithms were implemented with “train” function in the caret R package version 6.0-92 [42]. The method option in “train” function was set to “C5.0” and “rf” for implementing the C5.0 and RF algorithms, respectively. For all algorithms, 10-fold cross-validation (CV) was used to optimize the models. For the C5.0, both the tree and rule-based models with or without winnowing (i.e., a process of removing uninformative predictors) were evaluated and the best model was selected. The hyperparameter (the node size) in the RF algorithm by comparing the accuracy of models from different node sizes (1 to 10) was also optimized. The node size that resulted in the highest accuracy was selected for building the RF model.

The following metrics were used to evaluate the models: (a) accuracy (the percentage of correct predictions by the model), and kappa statistics/accuracy (an adjustment to predictive accuracy by accounting for the possibility of a correct prediction by chance alone). These metrics were computed from the “confusionMatrix” function in the caret R package version 6.0-92 [42]. In order to evaluate the performance of the proposed models, sensitivity and specificity were considered. Sensitivity refers to a proportion of days with sporangia concentrations >10 that correctly give, a positive result using the test in question. Specificity means the proportion of days with a concentration <10 sporangia/m^3^ that correctly give a negative result using the test in question.

Model performance was also assessed using receiver operating characteristics (ROC). A ROC curve is a graphical representation of sensitivity versus specificity for a binary classifying system. Thus, in ROC analysis, the area under the curve (AUC) represents the power of a parameter to discriminate between two classes. ROCs are used to evaluate the trade-off between true and false positives. A ROC curve shows the model’s prediction based on the positive class when the actual result is positive. Thus, in ROC analysis, the area under the curve represents the power of a parameter to discriminate between two classes. AUC values are interpreted as follows: <0.6 (no discrimination), 0.6–0.7 (poor), 0.7–0.8 (acceptable), 0.8–0.9 (excellent), >0.9 (outstanding). The ROC and AUC was computed with the ROCR package (version 1.0-11) [43].

## 3. Results

### 3.1. Crop Phenology during Period of Study

Planting dates in the five years took place between April and May (Table 1). The earliest planting year was 2017, while the latest planting year was 2020. Emergence occurred at 15 days, except in 2017, when it was delayed by 25 days. Flowering occurred in the 34–40 days after emerging, except in 2017, when it happened a few days earlier. Considering the days that elapsed since emergence, flowering occurred between days 29 (2017) and 40 (2019). The longer crop cycle (2018) reached the end of senescence at 106 days after emergence and the last two crop cycles, 2021 and 2020, were the shortest cycles and reached senescence at 78 and 83 days after emergence, respectively.

### 3.2. Overview of Weather Conditions by Phenological Stage

Specific mean values by phenological stage are presented in Table 2. Of the five years studied, the 2018 growing season was the wettest, with approximately 297 mm of water on 37 rainy days. In contrast, 2020 was the warmest and driest year. During the whole cycle, only 15 mm of rainfall was recorded in 7 days. In general, the foliar development (FD) was the stage with the mildest temperatures and the rainiest days, although not the most abundant in terms of rain amount. The average temperature in this phenological stage was 16.4 °C, with a mean of 10.6 days of rain. Additionally, this phenological stage was the one with the highest RH: 78.1% on average. On the contrary, during the senescence stage (SE), the lowest RH was recorded, with an average of 72.6%. In addition, in this final period, there were fewer rainy days and, consequently, less accumulated water (a mean of 4 days and 29.0 mm). The flowering (FL) was the stage with the highest average temperatures (19.4 °C) and mean RH (73.7%).

### 3.3. Daily Sporangia Concentration by Main Phenological Stage

The most abundant seasons in terms of daily sporangia concentration were 2018 and 2021, with 2271 and 1836 accumulated sporangia in each crop cycle, respectively (Table 3). Conversely, the year that stood out for its low concentration of sporangia was 2020, with a total of 36 sporangia in the whole cycle. In addition, in 2020, days with ARL were not found. In terms of phenological phase, the foliar development stage (FD) was the stage in which the highest daily sporangia concentrations were recorded. Additionally, the highest number of days with ARL was recorded during the foliar development stage (FD) except in 2017. In 2019, 100% of days with ARL was recorded during FD stage (Table 3).

### 3.4. Relationships between the Sporangia Concentration and the Meteorological Parameters

Spearman correlation coefficients were calculated between the meteorological variables and sporangia concentration. The highest positive and significant coefficients were found between the presence of sporangia of one and two previous days with daily sporangia concentration. Significantly negative coefficients between T and SR and the daily sporangia concentration were found (*p* < 0.05), while significantly positive coefficients between the sporangia and RH and IP were found. Wind and escape were positively correlated with sporangia from the second previous day until the fifth previous day. For LW, the correlation between sporangia concentration and the same-day recording (LW_0) was significantly positive, in contrast to the previous days (Table 4).

### 3.5. Machine Learning Algorithms to Predict the Daily Sporangia Risk Level

In order to develop a model to predict the ARL, ML algorithms (RF and C5.0) were applied using airborne daily sporangia and weather data. A binary prediction of 1 and 0 were designed (1: corresponded with days in which sporangia concentrations were equal or higher than 10 sporangia/m^3^; 0: days with concentrations lower than this value). Depending on the model, the ranking of the importance of the variables used for the developed model varied (Table 5). The C5.0 algorithm ranked the variables according to the usage ratio of each variable in the final result. The most decisive variables in the prediction performed by the C5.0 algorithm were Wind (_6, _2), the sporangia (_7, _1), T_7, LW_0, IP_0, SR_5 and RH (_7, _0). According to the RF algorithm, sporangia was the most influenced variable on the accuracy of model followed by SR_6, Escape_2 and IP_0. The sporangia of previous days (Sporangia_1) was ranked as the most important variable with a 36% decrease in accuracy.

Aerobiological variables related to the presence of sporangia on previous days were the most important variables in the prediction performed by the RF algorithm, coinciding with the higher significant Spearman correlation coefficients (*p* < 0.01) (Table 4). However, the weather variables selected (SR_6, Escape_2, IP_0) for the prediction by this algorithm were those with the highest significant Spearman correlation coefficients (*p* < 0.01). Most of the weather variables selected by the C5.0 algorithm (T_7, Wind_2, LW_0, IP_0) also had the highest significant correlation coefficients (*p* < 0.01) within their weather category.

The accuracy of two ML algorithms applied is shown in Table 6. The suitable node size was 1 for the RF model. For the C5.0 model, a tree-based model without winnowing was the best model. The results of prediction showed an accuracy of 87% and 85% for RF and C5.0, respectively. The Kappa values were 0.70 for RF and 0.65 for C5.0, which indicated that the RF results were less conditioned by chance than those provided by C5.0 algorithm. In both cases, the results were acceptable. Considering the parameter that indicates the percentage of success in the prediction of days with a sporangia concentration equal to or greater than 10 (Sensitivity), the C5.0 model was more accurate. On the contrary, the prediction of the models with the daily concentration of sporangia lower than the established threshold (specificity), RF model had a higher prediction of 0.92. However, despite using different relevance variables in the classification algorithms, the sporangia prediction accuracies were similar.

**Figure 2:**
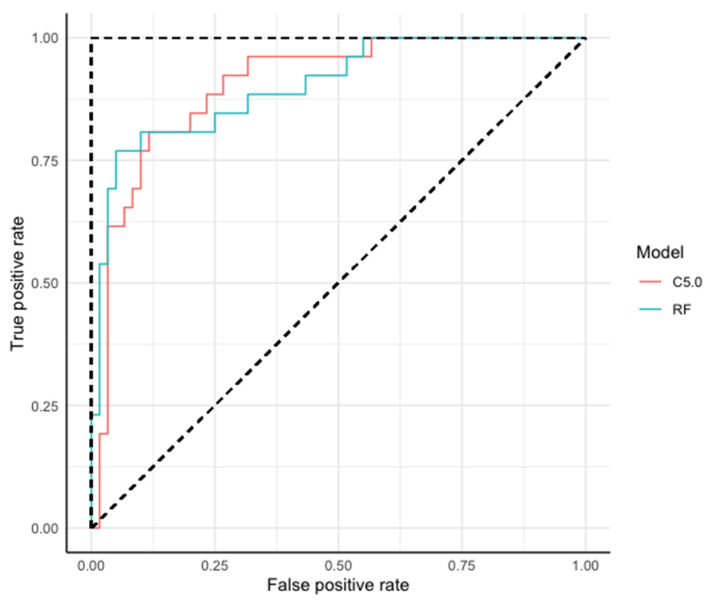
Receiver operating curve (ROC) for comparing the machine learning models random forest (RF) and C5.0 decision tree (C5.0). The broken lines in the ROC curve represent a classifier with no predictive value (diagonal line) and a perfect classifier (horizontal and vertical lines). ROC curves that are closer to the perfect classifier show a better model.

For the choice of the best algorithm, ROC curves were used to compare the ratio of false positives versus true positives (Figure 2). Both algorithms presented high and similar areas under the curve (AUC), 0.904 and 0.903 for the algorithm C5.0 and RF, respectively.

### 3.6. Checking Aerobiological Risk Level Prediction by Machine Learning Algorithms

**Figure 3:**
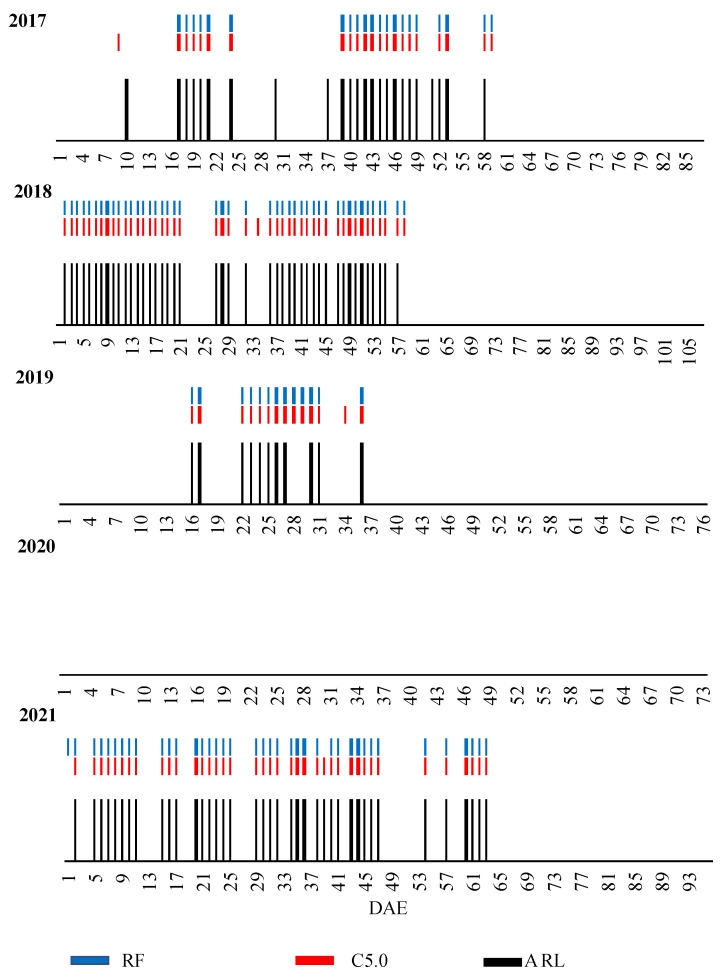
Comparison between prediction data by ML algorithms C5.0 and random forest (RF) and observed aerobiological risk level (ARL) by growing season. DAE: days after emergence.

The resulting predictions from each model algorithm with the observed SRL (aerobiological data) were compared. The prediction result of applying each of the algorithms to the sporangia level from the five growing seasons based on the SRL is shown in Figure 3. Overall, the algorithms made an accurate prediction in all five years of the study, even in the anomalous year of 2020. In this year, no day with a daily concentration of more than 10 sporangia/m^3^ was recorded and the algorithms did not predict risk for that year. The season with the highest number of days with a daily concentration above 10 sporangia/m^3^ were 2018 and 2021. In both high-risk and low-risk years, the two algorithms showed the prediction in agreement with the observed data (Figure 3).

Most of the positive predictions were shown in the first months of crop, coinciding with the phenological stages of FD and FL, from May to the beginning of July, except for in the year 2021, where the possibility of infection was extended to the beginning of August. In 2017, the C5.0 algorithm predicted the first risk day at the end of May a day in advance.

## 4. Discussion

The understanding of different factors that support the aerial dispersal of *P. infestans* for the correct prediction of late blight epidemics is crucial [7,8,10,14,15,34,44]. However, experimental studies under field conditions with the aim of predicting sporangia are limited. In this sense, the present study is one of the few that predicted airborne sporangia using the value of the inoculum quantified in the crop environment and at the same time, considering climatic factors. Several factors (e.g., wind, temperature, solar radiation, rain) could be used to forecast the outbreak of late blight [45]. This study focused on the main weather factors (T, RH, LW and wind) and derived variables (IP, SR and escape) with inoculum quantity to predict daily sporangia risk level that cause outbreaks of potato late blight. The results of this research support the importance of considering inoculum to make decisions of fungicides applications. This information combined with decision support system for late blight would improve the exactitude of warnings for a correct management of fungal treatments. The first application rate could be predicted before the onset of the disease in the field and consequently, the number of chemical applications would decrease.

According to previous research, linear regression models or neural network can predict with success disease behavior such as mycotoxin secretion in crops such as grapevine, rice and wheat or improve the ability of crop-growth monitoring [17,46,47,48,49,50]. In potato, the publications related with prediction of inoculum of late blight in air using ML algorithms are non-existent. For effective management of potato late blight, efforts must be made to slow the progress of the disease, especially by reducing the primary inoculum. In this sense, ML algorithms were applied because of their usefulness in managing large databases with multiples variables, images or spectra [17,18,19]. The performance of the models was evaluated as a binary classification system, categorizing the daily sporangia into two classes by aerobiological criteria (ARL) according to the daily sporangia concentration greater or less than 10 sporangia/m^3^. The C5.0 and RF algorithms were good and robust algorithms to predict days with high detached sporulation of *P. infestans*, resulting a ROC of 0.903 and 0.902, respectively. These ML algorithms provided good predictions in other potato pathogen, such as *Alternaria* spp. [37]. The ranking of the variables of importance by RF and C5.0 algorithms took the sporangia variables from previous days as the most influential to develop the prediction models. This suggests a strong influence of previous sporangia counts in predicting sporangia level at present, as also corroborated by the Spearman correlation test. A strong influence of sporangia of previous days in the presence of sporangia of present day was observed. This fact coincides with previous studies on *P. infestans* [10,15] and *Alternaria* [33,37,51,52] on potato crop, as well as *Botrytis cinerea* [53] and *Uncinula necator* [49] on vineyards. This trend emphasizes the need to continually monitor the airborne spores, as they will be needed to accurately predict future spread of spores.

To predict the behavior of *P. infestans*, it is important to know about crop season and geographical area [7]. Typically, the potato growing seasons in northwest Spain run from the beginning of May to the end of September. During this period, the temperature is the key weather factor for the growth of the potatoes. Throughout the growing season, spring rains are common and contribute to the development of the crop during the first few weeks. However, temperatures and high humidity also prove to be suitable for the presence of certain diseases for potato crop, such is the case of late blight. The production, dissemination and germination of *P. infestans* sporangia, as well as their penetration into host tissues are particularly influenced by mild temperatures and high relative humidity [1,7,8,10,14,15,34,44]. According to the Spearman correlation test, the coefficients between T and the sporangia presence were significantly negative. On the contrary, RH and LW variables showed a significantly positive relationship with the sporangia presence. Both algorithms agreed with IP and SR as the variables of higher importance. It is known that optimum temperatures for late blight epidemics are between 16 and 23 °C [6]. The IP variable combines the effect of T and RH under one variable, considering optimum values to late blight infection of 10–24 °C and RH > 88% [10]. In the studied area, mean temperatures between 16–21 °C were repeated during the whole period. In addition, rains and high humidity in the first months of crop development can explain higher sporangia concentration during foliar development stage. Under optimal temperature range to late blight epidemics, the lack of rain and dry weather could decrease the infection process and sporulation, as our results showed, with a decrease in rainy days and RH in flowering and senescence stages. Furthermore, it was shown that temperatures above 28 °C negatively affect sporangia production [54,55]. Thus, this could explain the lowest sporangia concentration trapped during senescence of five studied crop seasons.

The results agree with previous studies that showed changing dynamics of the *P. infestans* inoculum concentration during the potato growing season [9,10,14,28,34]. However, these studies focused on the prediction of late blight risk solely based on weather factors, while assuming the constant presence of inoculum [55,56,57]. Although the infection of potato plants with *P. infestans* is highly dependent on weather conditions, late blight epidemics cannot be explained exclusively by weather data [7,54]. Aerobiological information can be useful to avoid false alarms of infection risk when climatic conditions are favorable, but if there is no presence of inoculum in the air, there is no risk [34]. The variability in the sporangia concentrations during each phenological stage confirm that the presence of aerial inoculum in any place is not an unlimited factor. Thus, the climatic conditions, the local topography, the associated fungal host and the phenological state of the plant condition affect this variation [9,10,14,15,30]. Despite the scarcity of studies that considered quantification of the airborne inoculum, the present results agree with researchers that support its presence as essential to know the real late blight pressure in a particular area and to predict new reinfections [9,10,13,14,15,29,35,54]. However, for the success of the proposed methodology, several key factors are necessary: having several years of study, meteorological stations on the plot itself or in the vicinity of the monitored area, and specialized personnel for the extraction of aerobiological and meteorological data, as well as the correct treatment of algorithms with complex statistical methods. This type of research has great practical utility in the agricultural sector because it allows farmers to detect the infection before it manifests itself. Consequently, these professionals achieve greater effectiveness in the application of chemical treatments, and reduce investment in preventive treatments. The search for more environmentally sustainable agricultural solutions and tools to minimize the impact of these changing fungal diseases in recent years will have an impact on the economic value of the final product placed on the market. At the same time, it will favor food security and human health in the world.

## 5. Conclusions

The development of a simple and robust tool to forecast days with a high amount of inoculum capable of developing infection of late blight in potato crops can be useful for the sector. The information on the inoculum concentrations of *P. infestans* in the crop environment through aerobiological sampling was an important variable to consider in the development of ML techniques. The two ML algorithms applied (RF and C5.0) were accurate for the prediction of the aerobiological risk level (ARL) using meteorological parameters and inoculum of the pathogen from previous days. The different statistical treatments highlighted the influence of the sporangia from previous days. Furthermore, the most influential meteorological variables in the two validated ML algorithms were IP and SR. Therefore, it is interesting to integrate multiple meteorological variables and the presence of sporangia in the crop environment to guarantee the success of these prediction systems. This approach of using a classification model to forecast late blight risk may improve the accuracy of disease risk warning systems for potato plants in the study area. Thus, the utility of incorporating the inoculum present in the crop environment with weather variables in ML algorithms for monitoring of plant pathology in sustainable agricultural systems was demonstrated.

## Figures and Tables

**Table 1:** Dates of phenological stages in five studied crop seasons.

	Date (DAE)				
Stage	2017	2018	2019	2020	2021
Planting	22 April	15 May	16 May	26 May	22 May
Emerging	16 May (1)	1 June (1)	4 June (1)	10 June (1)	10 June (1)
Begining Flowering	13 June (29)	4 July (34)	13 July (40)	13 July (34)	14 July (35)
Start of senescence	19 July (65)	15 August (76)	17 August (75)	14 August (66)	7 August (59)
End of senescence	12 August (89)	14 September (106)	1 September (90)	31 August (83)	26 August (78)

DAE: days after emergence.

**Table 2:** Descriptive analysis of weather parameters by phenological stage during five studied crop seasons.

Season	Stage	T (°C)	RH (%)	Accumulated Rain (mm)	Rainy Days	Rainy Days (%)
2017	FD	16.7	76.0	21.4	6	31.6
	FL	19.6	72.9	155.8	10	52.6
	SE	17.7	69.6	84.2	3	15.8
2018	FD	16.3	82.7	123.4	20	54.1
	FL	19.3	76.3	121.4	9	24.3
	SE	19.2	69.8	52.0	8	21.6
2019	FD	15.7	76.9	42.8	13	48.1
	FL	18.5	76.5	33.6	10	37.0
	SE	18.9	72.4	4.0	4	14.8
2020	FD	17.0	74.2	8.2	2	28.6
	FL	21.4	65.4	4.2	2	28.6
	SE	16.6	77.9	2.6	3	42.9
2021	FD	16.3	80.7	68.0	12	54.5
	FL	17.9	77.4	38.4	8	36.4
	SE	19.2	73.2	2.4	2	9.1

Temperature (T) and relative humidity (RH) expressed in mean values.

**Table 3:** Sporangia information by phenological stage during the five studied crop seasons.

Season	Stage	Total *P. infestans* Sporangia	Days ≥ 10 Sporangia/m^3^	Days ≥ 10 Sporangia (%)
2017	FD	311	11	39.3
	FL	521	17	60.7
	SE	15	0	0
2018	FD	1723	25	55.6
	FL	509	20	44.4
	SE	39	0	0
2019	FD	481	11	100
	FL	37	0	0
	SE	5	0	0
2020	FD	28	0	0
	FL	7	0	0
	SE	1	0	0
2021	FD	1134	22	56.4
	FL	540	13	33.3
	SE	162	4	10.3

FD: foliar development; FL: flowering; SE: senescence.

**Table 4:** Spearman correlation coefficients between daily weather parameters and sporangia concentration until seven previous days.

Previous Days	T	RH	LW	Wind	SR	Escape	IP	Sporangia
0	−0.162 **	0.198 **	0.125 **	0.020	−0.179 **	0.030	0.258 **	1
1	−0.207 **	0.218 **	0.081	0.026	−0.169 **	0.062	0.259 **	0.669 **
2	−0.229 **	0.221 **	0.039	0.158 **	−0.156 **	0.182 **	0.256 **	0.555 **
3	−0.221 **	0.243 **	0.072	0.153 **	−0.180 **	0.162 **	0.247 **	0.485 **
4	−0.224 **	0.249 **	0.069	0.146 **	−0.185 **	0.158 **	0.254 **	0.427 **
5	−0.250 **	0.268 **	0.078	0.165 **	−0.198 **	0.178 **	0.252 **	0.430 **
6	−0.272 **	0.322 **	0.089	0.106 *	−0.247 **	0.114 *	0.247 **	0.402 **
7	−0.253 **	0.259 **	0.104 *	0.056	−0.200 **	0.066	0.224 **	0.421 **

* *p* <0.05; ** *p* <0.01. T: mean temperature; RH: mean relative humidity; LW: leaf wetness; SR: spore release; IP: infection pressure.

**Table 5:** Weather and sporangia variables in order of importance of selection according to ML algorithm.

C5.0 Algorithm		RF Algorithm	
Weather Variable	Usage Rate (%)	Weather Variable	Decrease in Accuracy (%)
Wind_2	100	Sporangia_1	36
Wind_6	100	Sporangia_3	14
Sporangia_7	100	Sporangia_2	13
Sporangia_1	100	Sporangia_4	11
T_7	100	Sporangia_5	10
LW_0	100	Sporangia_7	10
IP_0	100	Sporangia_6	9
SR_5	100	SR_6	6
RH_7	100	Escape_2	6
RH_0	99	IP_0	6

T: mean temperature; RH: mean relative humidity; LW: leaf wetness; SR: spore release; IP: infection pressure.

**Table 6:** Relevant information about predictions by machine learning algorithms.

Algoritm	Accuracy	Kappa	Sensitivity	Specificity
C5.0	0.85	0.65	0.81	0.87
RF	0.87	0.70	0.77	0.92

## Data Availability

Not applicable.
